# Vitrectomy with internal limiting membrane peeling versus inverted internal limiting membrane flap technique for macular hole-induced retinal detachment: a systematic review of literature and meta-analysis

**DOI:** 10.1186/s12886-017-0619-8

**Published:** 2017-11-28

**Authors:** Jing Yuan, Ling-Lin Zhang, Yu-Jie Lu, Meng-Yao Han, Ai-Hua Yu, Xiao-Jun Cai

**Affiliations:** Department of Ophthalmology, Zhongnan Hospital of Wuhan University, Wuhan, 430071 People’s Republic of China

**Keywords:** Macular hole-induced retinal detachment, Vitrectomy, Internal limiting membrane peeling, Inverted internal limiting membrane flap technique, Meta-analysis

## Abstract

**Background:**

To evaluate the effects on vitrectomy with internal limiting membrane (ILM) peeling versus vitrectomy with inverted internal limiting membrane flap technique for macular hole-induced retinal detachment (MHRD).

**Methods:**

Pubmed, Cochrane Library, and Embase were systematically searched for studies that compared ILM peeling with inverted ILM flap technique for macular hole-induced retinal detachment. The primary outcomes are the rate of retinal reattachment and the rate of macular hole closure 6 months later after initial surgery, the secondary outcome is the postoperative best-corrected visual acuity (BCVA) 6 months later after initial surgery.

**Results:**

Four studies that included 98 eyes were selected. All the included studies were retrospective comparative studies. The preoperative best-corrected visual acuity was equal between ILM peeling and inverted ILM flap technique groups. It was indicated that the rate of retinal reattachment (odds ratio (OR) = 0.14, 95% confidence interval (CI):0.03 to 0.69; *P* = 0.02) and macular hole closure (OR = 0.06, 95% CI:0.02 to 0.19; *P* < 0.00001) after initial surgery was higher in the group of vitrectomy with inverted ILM flap technique than that in the group of vitrectomy with ILM peeling. However, there was no statistically significant difference in postoperative best-corrected visual acuity (mean difference (MD) 0.18 logarithm of the minimum angle of resolution; 95% CI −0.06 to 0.43 ; *P* = 0.14) between the two surgery groups.

**Conclusion:**

Compared with ILM peeling, vitrectomy with inverted ILM flap technique resulted significantly higher of the rate of retinal reattachment and macular hole closure, but seemed does not improve postoperative best-corrected visual acuity.

## Background

Macular hole-induced retinal detachment (MHRD) often cause severe visual impairment, it occurs mainly in older with highly myopic eyes exist posterior staphyloma [1, 2]. The onset and progression of MHRD might be related to tangential traction due to the epiretinal membrane and posterior vitreous cortex complex, atrophy of the retinal pigment epithelium, disorder of internal limiting membrane (ILM), and vertical traction by the retina that cannot stretch after staphylomatous elongation of the globe [3–5]. Various surgical methods have been attempted to achieve improvement of anatomic and functional for MHRD, including macular buckling, pars plana vitrectomy, vitrectomy with scleral imbrications, vitrectomy with ILM peeling [6–9], among which vitrectomy with ILM peeling was thought to one of the most effective surgical procedures for MHRD, which achieved in a relatively high initial retinal reattachment rates ranging from 42% to 93%, but a relatively poor initial macular hole closure rates ranging from 10% to 70% [10–13]. An open macular hole in the eye with highly myopic induce the risk of recurrent retinal detachment, which may injure central vision in the future [14]. In addition, The peeling of the ILM seems contribute little to the improvement of visual acuity for MHRD [15, 16]. Recently, The inverted ILM flap technique was first described by Michalewska et al [17] has been extend used for treating MHRD, contributed to a relatively high macular hole closure rates [18, 19]. Is the inverted ILM flap technique a preferable option for MHRD? There have been comparisons of the inverted ILM flap technique and ILM peeling for MHRD, but the results are contradictory. Thus, we conducted a meta-analysis to compare the effects of vitrectomy with ILM peeling vs vitrectomy with inverted ILM flap technique for MHRD, the rate of retinal reattachment, the rate of macular hole closure 6 months later after initial surgery and the postoperative BCVA 6 months later after initial surgery are used to compare the effects of the two surgery groups.

## Methods

### Search strategy

Pubmed, Cochrane Library and Embase were cautiously searched, the terms used for systematic search were “internal limiting membrane peeling”, “inverted internal limiting membrane flap technique”, “ILM flap”, “inverted internal limiting membrane insertion”, “internal limiting membrane repositioning”, these terms were connected with “or”. We also manually collected reference lists of original studies and review articles, there were no language or publication year restrictions, the final search was performed on May 2017. The titles and abstracts were assessed, and studies that did not compare surgical outcomes between patients of MHRD who had vitrectomy with ILM peeling and those who had vitrectomy with inverted ILM flap technique were excluded. Studies included cases with both macular holes (MHs) and peripheral breaks, the outcomes and parameters of patients were not clearly reported were also excluded. Full reports were retrieved and assessed for eligibility after the initial screening.

### Eligible criteria

All publications obtained from Internet-based searches were screened by predefined selection criteria. Eligible studies were randomized or nonrandomized studies among patients who had MHRD, that compared the rate of retinal reattachment, the rate of macular hole closure, best-corrected visual acuity after initial surgery between patients who had vitrectomy with ILM peeling and those who had vitrectomy with inverted ILM flap technique. Two reviewers (Y.L. And L.Z) completed the assessment of search results to identify included studies.

### Data extraction

The data on papers were independently extracted and rechecked by two reviewers (J.L. And L.Z). Any disagreement regarding eligibility during the extraction was resolved by discussion. The extracted information from each study included the rate of retinal reattachment and macular hole closure, the best-corrected visual acuity, first author, year of publication, the study design, number of the patients, age, surgical procedures, length of follow-up, baseline characteristics of the patients such as axial length, status of posterior staphyloma, lens status, dye used to visualize ILM, type of tamponade.

### Qualitative and risk bias assessment

The quality of each included study was assessed according to the methodological index for non-randomized studies (MINORS) [20]. It specifically designed for non-randomized noncomparative and comparative studies. This validated index involves 12 items, items are scored as 0 (not reported), 1 (reported but inadequate) and 2 (reported and adequate). 24 is the maximum score for comparative studies. We cautiously evaluated each study with a quality score and the score of 12 or more indicated a higher quality study. Visual inspection of the funnel plots and assess Egger’s regression test quantitatively were used to identify any potential publication bias, the Egger’s regression test would not be conducted if the included studies < 10 cases.

### Statistical analysis

RevMan 5.3 software was used for statistical analyses. To compare the rate of retinal reattachment and macular hole closure, we estimated odds ratios (ORs) and 95% CIs using Mantel–Haenszel method in a fixed effects model. For the evaluation of BCVA, the mean differences (MDs) of preoperative and postoperative measurements between the two methods were compared using weighted MDs and 95% CIs estimated random effects models. The continuous data such as median and range values in included studies were converted to the mean and standard deviation by using the method reported by Hozo et al. [21] Heterogeneity was assessed by calculating I^2^ and the chi-square statistic. I^2^>50% was considered to indicate considerable heterogeneity among the studies included in a meta-analysis. P<0.05 was considered statistically significant on the test for overall effect.

## Results

### Selection of studies and quality assessment

**Fig. 1:**
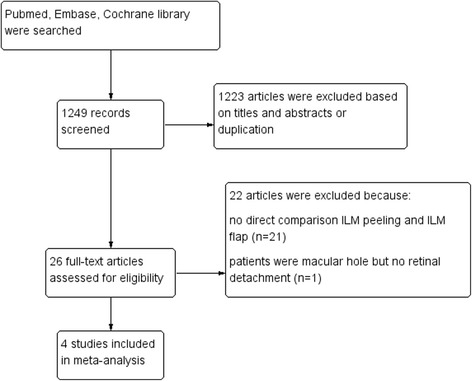
Selection of studies

Totally, 1249 articles were initially identified by the electronic searches. After screening all titles and abstracts of potentially relevant articles, most of these articles were excluded because of duplicates, case reports, review and other study subjects irrelevant to our target. After reading carefully of remaining potentially relevant articles, Finally, a total of 4 studies, were selected for the meta-analysis (Fig. 1) [22–25]. The methodologic quality of the included trials is explained comprehensively in Table 1. The scores of the included studies ranged from 16 to 18, In general, the quality of the studies was moderate to good. All data were analyzed in accordance with intention-to-treat principle.

**Table 1:** MINORS for assessing quality of included studies

Methodological item for non-randomized studies	Matsumura et al [22]	Baba et al [23]	Sasaki et al [24]	Chen et al [25]
1. A clearly stated aim	2	2	2	2
2. Inclusion of consecutive patients	2	2	2	2
3. Prospective collection of data	0	0	0	0
4. Endpoints appropriate to the aim of the study	2	2	2	2
5. Unbiased assessment of the study endpoint	0	0	0	0
6. Follow-up period appropriate to the aim of the study	2	2	2	2
7. Loss to follow up less than 5%	2	2	2	2
8. Prospective calculation of the study size	0	0	0	0
9. An adequate control group	2	2	2	2
10. Contemporary groups	0	0	0	0
11. Baseline equivalence of groups	2	2	2	2
12. Adequate statistical analyses	2	2	2	2
Total score	16	16	16	18

### Characteristics and baseline of the included studies

The characteristics of the included studies are list in Table 2. In total, 98 eyes included with retinal detachment resulting from macular hole. The number of eyes that had vitrectomy with ILM peeling was 52, and the number of eyes that underwent vitrectomy with inverted ILM flap technique was 46. Four studies are retrospective studies. The shortest follow-up duration was 6 months in 3 studies [23–25], the follow-up duration was 12 months in 1 study [22]. 25-gauge vitrectomy was performed in 3 studies [22–24], 23-gauge vitrectomy was performed in 1 study [25]. To visualize ILM, brilliant blue G was used in 3 studies [22–24], indocyanine green (ICG) was used in 1 study [25]. C_3_F_8_ gas tamponade was applied in 4 studies, SF_6_ gas tamponade was used in 2 studies [22, 24], Silicone oil tamponade was used in 1 study [22]. In order to minimize the effect of Lens status on the postoperative BCVA. Standard phacoemulsification and intraocular lens implantation was performed on all phakic eyes prior to vitrectomy in two groups in 1 study [22]. Phacoemulsification with intraocular lens implantation was performed in eyes that had cataracts in two groups in 2 studies [23, 24]. Lens status kept same preoperative and postoperative in 1 study [25]. All the patients included in those 4 studies who had gas as cavity tamponade were asked to maintain a facedown or prone position postoperatively for at least 5 days. The baseline characteristics of each included study, such as Lens status and axial length were found to be equivalent between the two groups in all 4 studies.

**Table 2:** Characteristics of studies included in this meta-analysis

Study	year	country	Study type	Group	NO. Of eyes	Age(yr)	Duration of symptoms (days)	Axial length (mm)	Dye for ILM stained	Tamponade agents
Matsumura [22]	2016	Japan	Retro	ILM peeling	12	75.30 ± 8.70	NR	30.40 ± 1.60	brilliant blue G	C_3_F_8_/SF_6_/Silicone oil
				ILM flap	10	67.70 ± 9.70	NR	28.40 ± 2.20	brilliant blue G	C_3_F_8_/SF_6_/Silicone oil
Baba et al [23]	2017	Japan	Retro	ILM peeling	11	69.75 ± 91.70	NR	30.17 ± 1.68	brilliant blue G	C_3_F_8_
				ILM flap	10	73.00 ± 96.67	NR	28.98 ± 1.31	brilliant blue G	C_3_F_8_
Sasaki et al [24]	2017	Japan	Retro	ILM peeling	9	66.00 ± 12.5	79.1 ± 66.5	31.10 ± 1.95	brilliant blue G	C_3_F_8_
				ILM flap	6	75.00 ± 6.40	47.6 ± 40.6	30.47 ± 2.57	brilliant blue G	C_3_F_8_
Chen et al [25]	2016	Taiwan	Retro	ILM peeling	20	60.53 ± 8.78	161.60 ± 136.2	29.35 ± 1.88	ICG	C_3_F_8_
				ILM flap	20	62.06 ± 8.90	587.0 ± 1032.53	28.40 ± 1.94	ICG	C_3_F_8_

### The rate of retinal reattachment 6 months later after initial surgery

**Fig. 2:**
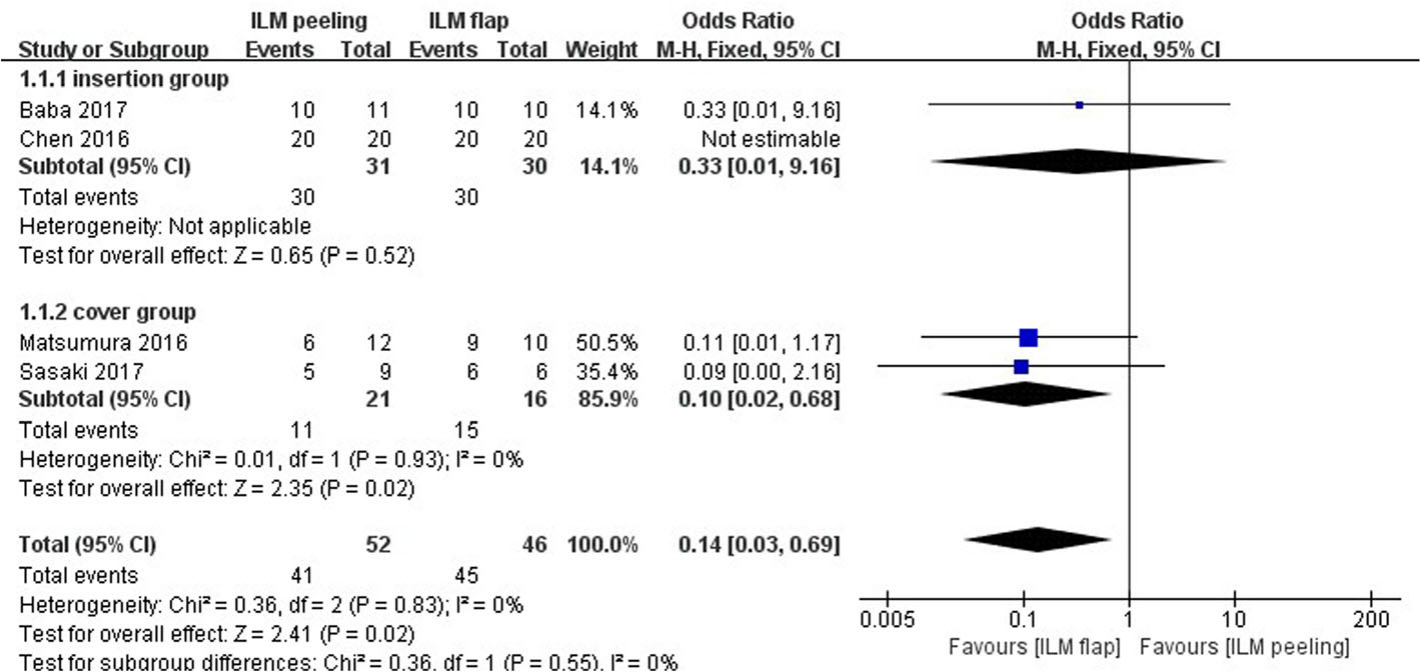
Meta-analysis comparing the rate of retinal reattachment between ILM peeling and ILM flap groups 6 months later after initial surgery. Insertion group represent the inverted ILM flap was pushed into the macular hole; Cover group represent the inverted ILM flap was placed over the macular hole. Odds ratio was calculated

Figure 2 shows the results of the meta-analysis comparing the rate of retinal reattachment after initial surgery between the group of vitrectomy with ILM peeling and the group of vitrectomy with inverted ILM flap technique. 4 studies of 98 eyes were included in this analysis. The rate of retinal reattachment after initial surgery had been evaluated after at least 6 months in all eyes. In total, the rate of retinal reattachment after initial surgery was 82.0% (41/52 eyes) in the vitrectomy with ILM peeling group and 97.8% (45/46 eyes) in the vitrectomy with inverted ILM flap technique group. The rate of retinal reattachment in two groups were relatively high, and the rate of retinal reattachment in the vitrectomy with inverted ILM flap technique group was still significantly higher than that in the vitrectomy with ILM peeling group (OR = 0.14, 95% CI: 0.03 to 0.69; *P* = 0.02). There was no statistical heterogeneity between the two groups (heterogeneity I^2^ = 0%).

### The rate of macular hole closure 6 months later after initial surgery

**Fig. 3:**
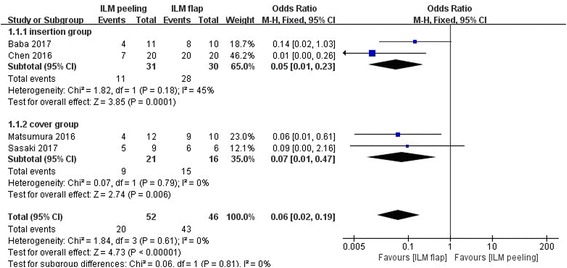
Meta-analysis comparing the rate of macular hole closure between ILM peeling and ILM flap groups 6 months later after initial surgery. Insertion group represent the inverted ILM flap was pushed into the macular hole; Cover group represent the inverted ILM flap was placed over the macular hole. Odds ratio was calculated

The rate of macular hole closure after initial surgery was reported in all 4 studies including 98 eyes, and the rate of macular hole closure after initial surgery had also been evaluated after at least 6 months in all eyes. There was no statistical heterogeneity between the studies (heterogeneity I^2^ = 0%). Overall, the rate of macular hole closure after initial surgery was 38.5% (20/52 eyes) in the vitrectomy with ILM peeling group and 93.5% (43/46 eyes) in the vitrectomy with inverted ILM flap technique group. The rate of macular hole closure after initial surgery was significantly higher in the vitrectomy with inverted ILM flap technique group than that in the vitrectomy with ILM peeling group (OR = 0.06, 95% CI:0.02 to 0.19; *P* < 0.00001; Fig. 3).

### Preoperative best-corrected visual acuity

**Fig. 4:**
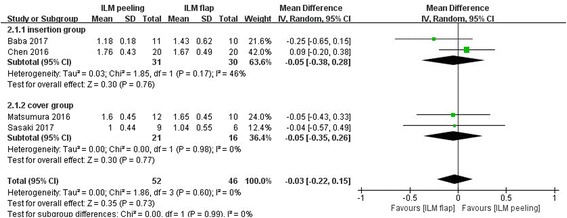
Meta-analysis comparing preoperative BCVA between ILM peeling and ILM flap groups. Insertion group represent the inverted ILM flap was pushed into the macular hole; Cover group represent the inverted ILM flap was placed over the macular hole. Mean difference was calculated by logarithm of the minimum angle of resolution

Figure 4 shows the results of the meta-analysis comparing preoperative BCVA between the group of vitrectomy with ILM peeling and the group of vitrectomy with inverted ILM flap technique. This analysis included all 4 studies with 98 eyes. There was no significant difference between two surgical approaches in preoperative BCVA (MD -0.03 logarithm of the minimum angle of resolution; 95% CI-0.22 to 0.15; *P* = 0.73). There was no statistical heterogeneity between the studies (heterogeneity I^2^ = 0%).

### Postoperative best-corrected visual acuity 6 months later after initial surgery

**Fig. 5:**
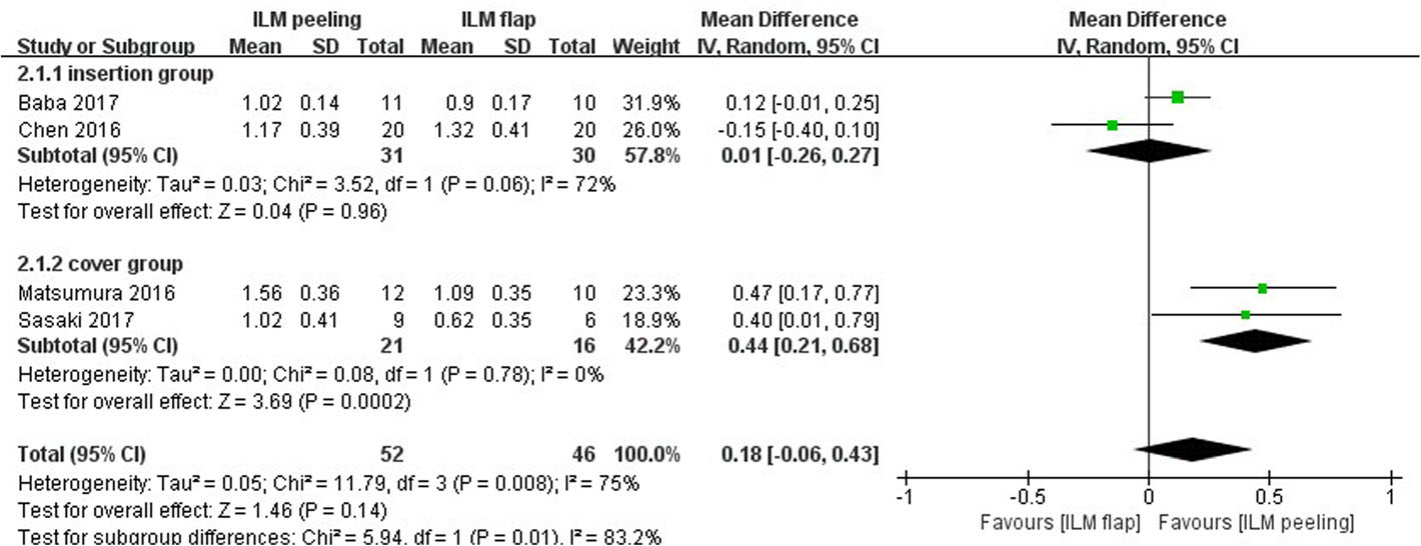
Meta-analysis comparing postoperative BCVA between ILM peeling and ILM flap groups 6 months later after initial surgery. Insertion group represent the inverted ILM flap was pushed into the macular hole; Cover group represent the inverted ILM flap was placed over the macular hole. Mean difference was calculated by logarithm of the minimum angle of resolution

Figure 5 shows the results of the meta-analysis comparing postoperative BCVA between the group of vitrectomy with ILM peeling and the group of vitrectomy with inverted ILM flap technique. For this analysis, all 4 studies with 98 eyes were included. There was no statistically significant difference in best-corrected visual acuity (MD 0.18 logarithm of the minimum angle of resolution; 95% CI -0.06 to 0.43; *P* = 0.14) between the group of vitrectomy with inverted ILM flap technique and the group of vitrectomy with ILM peeling. Heterogeneity was relatively high (heterogeneity I^2^ = 75%), after carefully read the included four studies, the included studies were considered clinically similar, a random effects model was used.

**Fig. 6:**
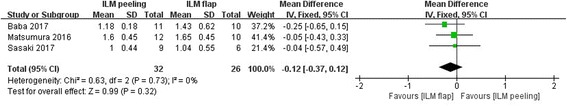
Meta-analysis comparing preoperative BCVA between ILM peeling and ILM flap groups that only Brilliant Blue G was used to visualize ILM during surgery. Mean difference was calculated by logarithm of the minimum angle of resolution

**Fig. 7:**
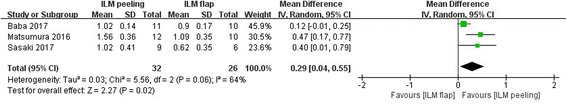
Meta-analysis comparing postoperative BCVA between ILM peeling and ILM flap groups that only Brilliant Blue G was used to visualize ILM during surgery 6 months later after initial surgery. Mean difference was calculated by logarithm of the minimum angle of resolution

In order to eliminate the effect of ICG use on postoperative BCVA, a meta-analysis of the subgroup of three studies in which only Brilliant Blue G was used to visualize ILM during surgery was performed. However, the postoperative BCVA (MD 0.29 logarithm of the minimum angle of resolution; 95% CI 0.04 to 0.55; *P* = 0.02) was significantly better in the group of vitrectomy with inverted ILM flap than that in the group of vitrectomy with ILM peeling. The included three studies were considered clinically similar, a random effects model was used (heterogeneity I^2^ = 64%) (Figs. 6, 7).

### Testing for publication bias

**Fig. 8:**
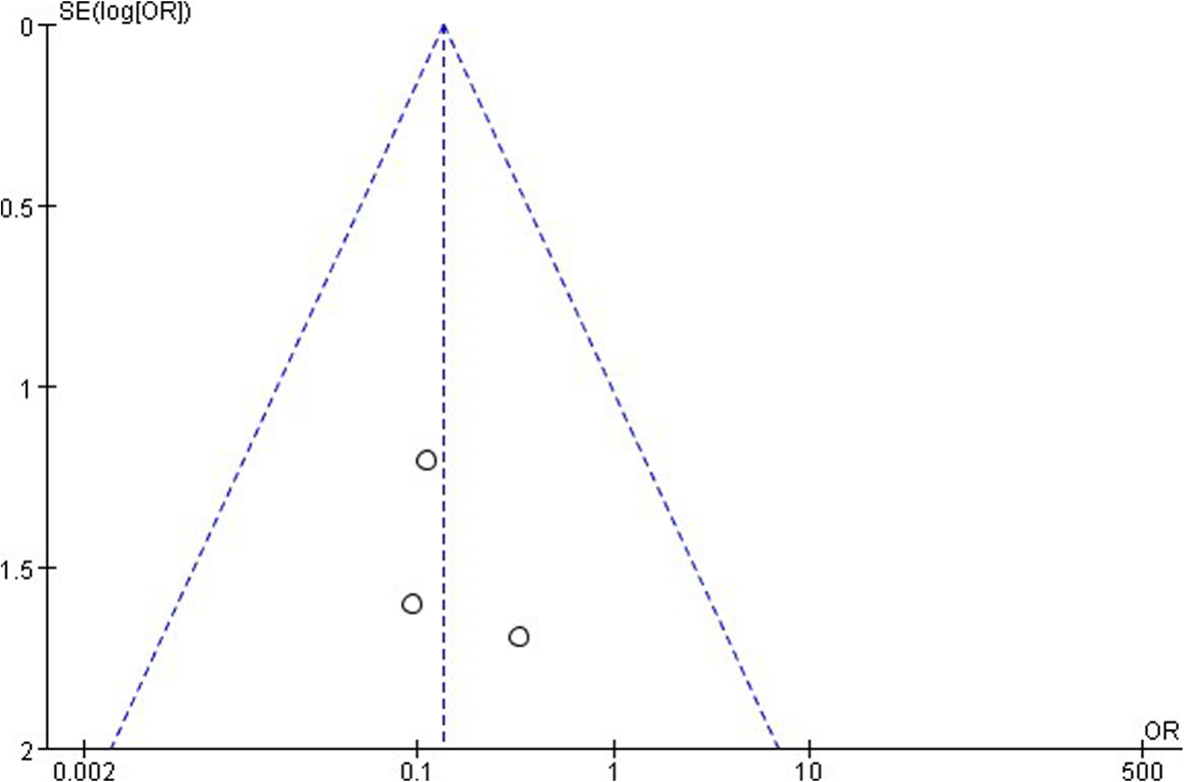
A funnel plot for the result from the studies comparing the rate of retinal reattachment showing no significant publication bias. SE = standard error, OR = odds ratio

**Fig. 9:**
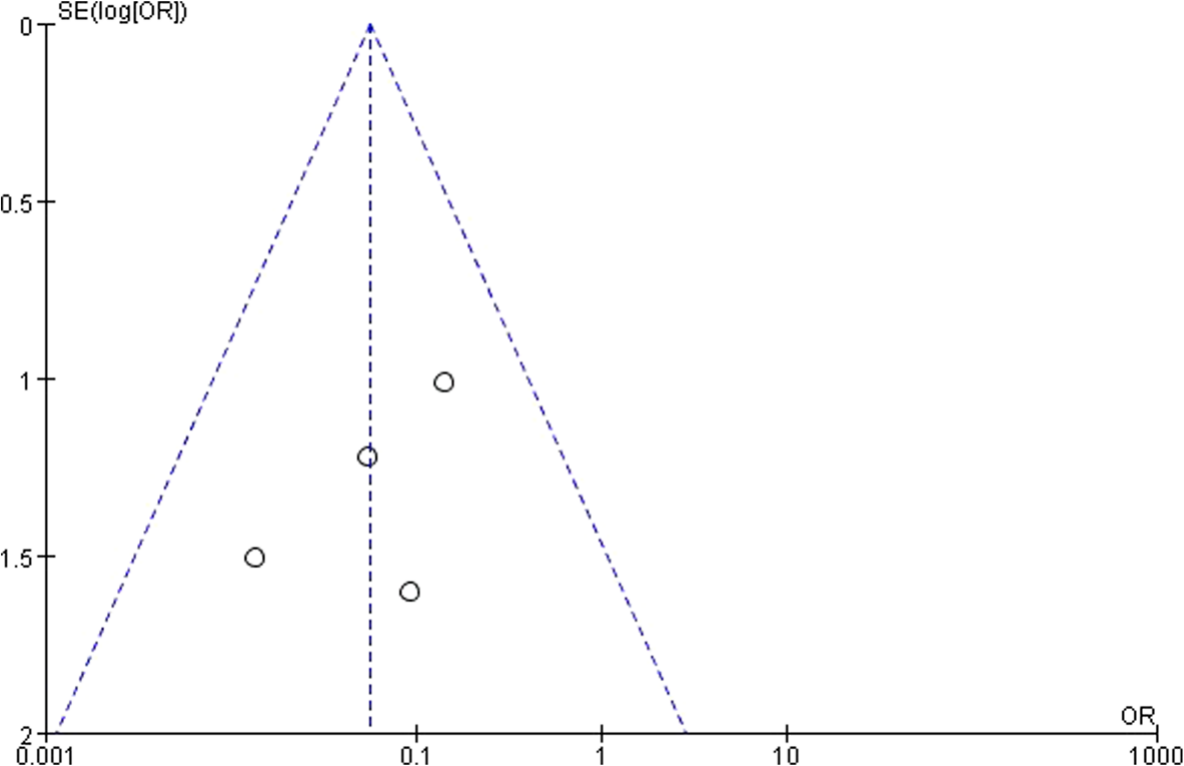
A funnel plot for the result from the studies comparing the macular hole closure showing no significant publication bias. SE = standard error, OR = odds ratio

**Fig. 10:**
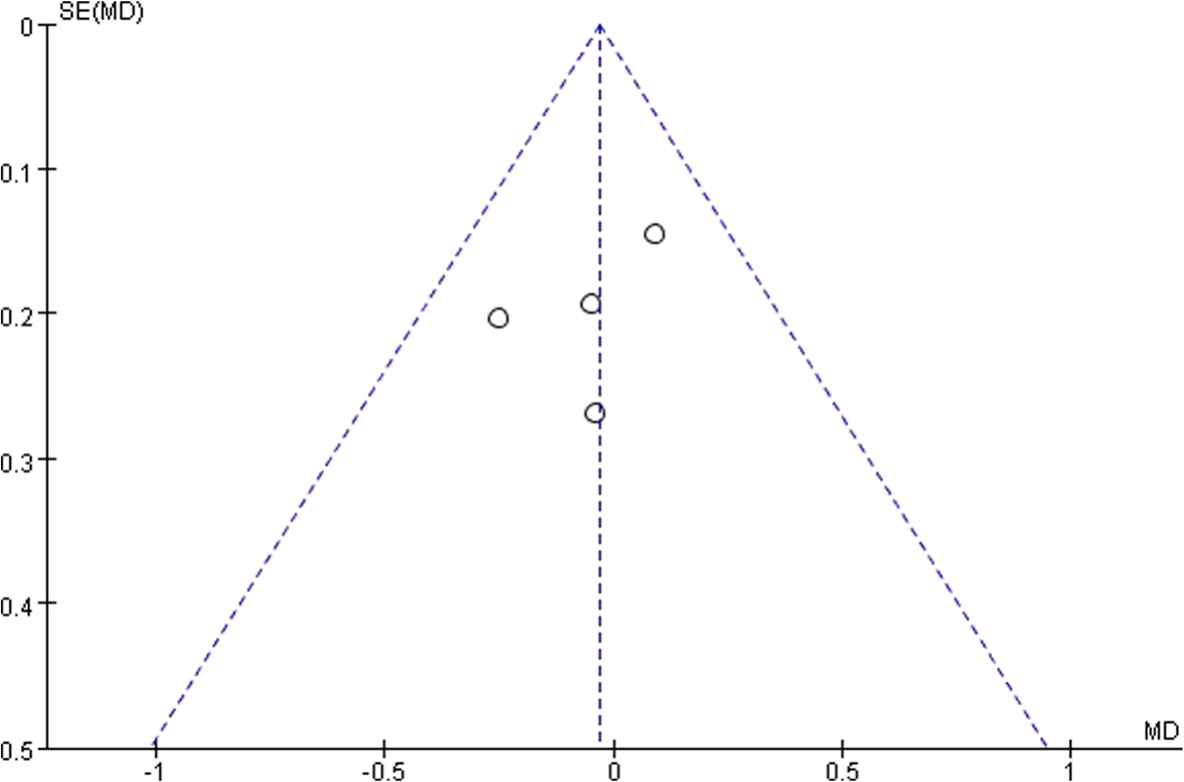
A funnel plot for the result from the studies comparing the preoperative BCVA showing no significant publication bias. SE = standard error, MD = mean difference

Three funnel plots of the retinal reattachment, the macular hole closure rate, the preoperative BCVA in including studies demonstrated symmetry, which all indicated no serious publication bias (Figs. 8, 9, 10).

## Discussion

In consider of macular hole-induced retinal detachment is relatively uncommon, it seems difficult to perform large scale studies or randomized studies to compare the effect of two surgery groups. We conducted this systematic review of literature and meta-analysis to summarize current evidence and compare the effect of vitrectomy with inverted ILM flap technique versus vitrectomy with conventional ILM peeling for MHRD. The result of this meta-analysis indicated that the rate of retinal reattachment and macular hole closure after initial surgery was significantly higher in the surgery of vitrectomy with inverted ILM flap than that in the surgery of vitrectomy with ILM peeling. However, the postoperative BCVA were similar between the two groups. Compared with conventional ILM peeling, vitrectomy with inverted ILM flap technique seems to improve the anatomical results rather the functional results for MHRD.

The rate of retinal reattachment (OR 0.14) and macular hole closure (OR 0.06) after initial surgery was significantly lower in the group of vitrectomy with ILM peeling than that in the group of vitrectomy with inverted ILM flap technique. The ILM peeling allows total removal of the cortical vitreous, releases the macular traction and stretches the retina, which may promote closure of macular hole and reattachment of retinas in MHRD [26]. However, MHRD itself often accompanied by posterior staphyloma, scleral elongation, atrophy of retina and choroid, which made the ILM peeling cannot eliminate the retinal tension completely and compensate for retinal shortening, then affect the retinal reattachment and macular hole closure. The relatively low rate of macular hole closure in the conventional ILM peeling group seem supported the hypothesis mentioned above. The inverted ILM flap technique was effective for the treatment of idiopathic large MHs and myopic MHs through filling the hole after stimulation of glial cell proliferation, thereby enhancing retinal reattachment and macular hole closure [17, 27]. In histopathologic findings, the ILM serves as a scaffold for gliosis, that the proliferated glial cells may fill the macular hole and compensate for retinal shortening [28]. It may be explained to the relatively high rate of macular hole closure in the inverted ILM flap technique group. The low closure rate for MHs has long been a problem in MHRD. Unclosed MH after surgery may cause redetachment of the retina in the future. Therefore, the inverted ILM flap technique will reduce the risk of retinal redetachment and the need for reoperation. Compared with conventional ILM peeling, inverted ILM flap technique can improve the anatomical results for MHRD.

It may contrary to expectation, the rate of macular hole closure after initial surgery was significantly higher in the group of vitrectomy with inverted ILM flap technique than that in the group of vitrectomy with ILM peeling (OR 0.06). However, there was no clinically or statistically significant difference in postoperative BCVA between the two groups of eyes treated by different surgical strategies (MD 0.18). Lam et al [8] and Ikuno et al [14] reported that postoperative BCVA was significantly better in eyes with macular hole closure than in those without closure after vitrectomy. Nishimura et al [29] and Nadal et al [30] reported no significant difference in postoperative BCVA between the eyes with and those without macular hole closure. The association between macular hole closure and BCVA after vitrectomy in eyes with MHRD were reported in several documents and the results were contradictory. It is believed that irreversible damage of the foveal photoreceptor has already occurred before the initial vitrectomy in MHRD eyes, such as chorioretinal atrophy and posterior staphyloma. The results of meta-analysis support the hypothesis that there is no significant difference in postoperative BCVA between the eyes with and those without macular hole closure for MHRD. Michalewska et al [17] found the inverted ILM flap technique improves not only the macular hole closure rate but also postoperative visual acuity. They hypothesized that the inverted ILM flap technique induces glial cell proliferation, then producing an environment for the photoreceptors to assume new positions in direct proximity to the fovea, and improves postoperative visual acuity. The microstructural changes of retina after the two surgeries have been studied, Modi et al [31] found that ganglion cell and inner plexiform layer were the only layers to show thinning in medial and temporal sectors signifying the fact that these are the layers which bear maximum brunt of the maneuver and show significant damage over a large area around the fovea as a result of ILM peeling. Hayashi et al [32] found that the foveal photoreceptor layer may be destroyed and not recoverable although the retina is reattached after surgical closure by the inverted ILM flap technique. The results of this meta-analysis that no significant difference in visual acuity between the ILM-peeling group and the inverted ILM flap technique group may support their hypothesis that the foveal structure of the retinal outer layer may be unrecoverable after vitrectomy for MHRD in highly myopic eyes.

The meta-analysis of the subgroup of three studies [22–24] in which only Brilliant Blue G was used to visualize ILM during surgery showed that the postoperative BCVA (MD 0.29) was significantly better in the group of vitrectomy with inverted ILM flap technique than that in the group of vitrectomy with ILM peeling. Compared with conventional ILM peeling, it seems that inverted ILM flap technique can improve the functional results for MHRD when Brilliant Blue G was used. It can also be speculated that the potential damage to the retinal pigment epithelium and neurosensory retina caused by cytotoxicity of vital dyes by introducing ILM tissue into the macular hole should be addressed. The cytotoxicity of vital dyes could be the potential reason for no difference in postoperative BCVA between the two surgery groups and the eyes with closed macular hole and those with macular holes remaining open.

According to the original report of the inverted ILM flap technique, the inverted ILM flap was placed over the macular hole to cover the surface of the hole [17]. Lai et al [33] reported that the inverted ILM flap was pushed gently into the macular hole so called internal limiting membrane repositioning, they achieved 96% closure rates of macular hole after initial surgery. In this meta-analysis, two studies conducted the inverted ILM flap technique according to the original report that the inverted ILM flap was placed over the macular hole to cover the surface of the hole [22, 24]. The other two studies conducted the inverted ILM flap technique that the inverted ILM flap was pushed gently into the macular hole to fill the entire hole instead of covering the hole [23, 25]. In the subgroup analysis, when the inverted ILM flap was pushed gently into the macular hole, the rate of retinal reattachment is similar between the two surgery groups (*p* = 0.52), the rate of macular hole closure is still significantly higher in the inverted ILM flap technique group (*p* = 0.0001), there was no statistically significant difference in postoperative BCVA between the two surgery groups(*p* = 0.96); when the inverted ILM flap was placed over the macular hole, the rate of retinal reattachment and the macular hole closure were significantly higher in the inverted ILM flap technique group(*p* = 0.02; *p* = 0.006 respectively), the postoperative BCVA was significantly better in the inverted ILM flap technique group (*p* = 0.0002). In consideration of potential toxicity of the ICG used in 1 study [25], In the inverted ILM flap technique that whether the inverted ILM flap should be placed over the macular hole to cover the surface of the hole or should be pushed gently into the macular hole is controversial. An inverted ILM insertion may decrease the risk of the ILM flap reverting to the previous state and tearing off during surgery, especially during fluid–air exchange. However, the inverted ILM insertion may disturb the migration of glial cells and visual cells and interfere with the recovery of the retinal layer. Gasini et al [34] conducted a study that whether surgical manipulation steps of the ILM flap are mandatory to obtain satisfactory outcomes for the repair of large stage IV idiopathic macular hole using the inverted ILM flap technique. And found that Internal limiting membrane finishing, tucking, and massage may not be required to obtain surgical success. Michalewska et al [35] reported that reducing the area of ILM peeling in the inverted ILM flap technique is as effective as the classic inverted ILM flap technique for the repair of large Stage IV macular holes. May these modified inverted ILM flap techniques will be extend used for treating MHRD in the future, to simplify the procedures of the inverted ILM flap technique, and improve the anatomical and functional results for MHRD.

The results of the meta-analysis should be interpreted with caution because of several limitations. First, In this meta-analysis, a small number of patients in included studies had a history of vitrectomy. Mylonas et al [36] reported that patients with previous vitrectomy and membrane and ILM peeling often develop macular edema after successful cataract surgery. Another limitation is that all the studies available for this meta-analysis were retrospective studies and the number of included patients is relatively small, we carefully evaluated patient selection, allocation, procedure equality, and definitions of outcome measures to select eligible studies for the present meta-analysis. However, significant heterogeneity among the studies was detected when we examined postoperative BCVA. Next one, converting non-normally distributed statistics (median and range) to normally distributed statistics (mean and SD) and publication bias that usually existed in meta-analysis based on published studies may be a cause of bias in this meta-analysis, in spite of the funnels plot showed there were no serious publication bias.

## Conclusion

In conclusion, apart from the limitations, Compared with ILM peeling, inverted ILM flap technique is considered to significantly improve the rate of retinal reattachment and macular hole closure after initial surgery, without significant adverse effects on postoperative BCVA. Larger randomized and prospective studies would be necessary to further confirm the effects of the inverted ILM flap technique for MHRD.

## Abbreviations

BCVA: best-corrected visual acuity
CI: confidence interval
ICG: indocyanine green; SE: standard error
ILM: internal limiting membrane
MD: mean difference
MHRD: macular hole-induced retinal detachment
MHs: macular holes
MINORS: methodological index for non-randomized studies
OR: odds ratio

## References

[1] Morita H, Ideta H, Ito K, Yonemoto J, Sasaki K, Tanaka S (1991). Causative factors of retinal detachment in macular holes. Retina.

[2] Akiba J, Konno S, Yoshida A (1999). Retinal detachment associated with a macular hole in severely myopic eyes. Am J Ophthalmol.

[3] Ishida S, Yamazaki K, Shinoda K, Kawashima S, Oguchi Y (2000). Macular hole retinal detachment in highly myopic eyes: ultrastructure of surgically removed epiretinal membrane and clinicopathologic correlation. Retina.

[4] Ichibe M, Yoshizawa T, Murakami K (2003). Surgical management of retinal detachment associated with myopic macular hole: anatomic and functional status of the macula. Am J Ophthalmol.

[5] Kadonosono K, Yazama F, Itoh N (2001). Treatment of retinal detachment resulting from myopic macular hole with internal limiting membrane removal. Am J Ophthalmol.

[6] Ando F, Ohba N, Touura K, Hirose H (2007). Anatomical and visual outcomes after episcleral macular buckling compared with those after pars plana vitrectomy for retinal detachment caused by macular hole in highly myopic eyes. Retina.

[7] Uemoto R, Yamamoto S, Tsukahara I, Takeuchi S (2004). Efficacy of internal limiting membrane removal for retinal detachments resulting from a myopic macular hole. Retina.

[8] Lam RF, Lai WW, Cheung BT (2006). Pars plana vitrectomy and perfluoropropane (C3F8) tamponade for retinal detachment due to myopic macular hole: a prognostic factor analysis. Am J Ophthalmol.

[9] Fujikawa M, Kawamura H, Kakinoki M (2014). Scleral imbrication combined with vitrectomy and gas tamponade for refractory macular hole retinal detachment associated with high myopia. Retina.

[10] Oie Y, Emi K, Takaoka G, Ikeda T (2007). Effect of indocyanine green staining in peeling of internal limiting membrane for retinal detachment resulting from macular hole in myopic eyes. Ophthalmology.

[11] Ripandelli G, Coppe AM, Fedeli R, Parisi V, D'Amico DJ, Stirpe M (2001). Evaluation of primary surgical procedures for retinal detachment with macular hole in highly myopic eyes: a comparison [corrected] of vitrectomy versus posterior episcleral buckling surgery. Ophthalmology.

[12] Lim LS, Tsai A, Wong D (2014). Prognostic factor analysis of vitrectomy for retinal detachment associated with myopic macular holes. Ophthalmology.

[13] Seike C, Kusaka S, Sakagami K, Ohashi Y (1997). Reopening of macular holes in highly myopic eyes with retinal detachments. Retina.

[14] Ikuno Y, Sayanagi K, Oshima T (2003). Optical coherence tomographic findings of macular holes and retinal detachment after vitrectomy in highly myopic eyes. Am J Ophthalmol.

[15] Wei Y, Wang N, Zu Z (2013). Efficacy of vitrectomy with triamcinolone assistance versus internal limiting membrane peeling for highly myopic macular hole retinal detachment. Retina.

[16] Su J, Liu X, Zheng L, Cui H (2015). Vitrectomy with internal limiting membrane peeling vs no peeling for macular hole-induced retinal detachment (MHRD): a meta-analysis. BMC Ophthalmol.

[17] Michalewska Z, Michalewski J, Adelman RA, Nawrocki J (2010). Inverted internal limiting membrane flap technique for large macular holes. Ophthalmology.

[18] Kinoshita T, Onoda Y, Maeno T. Long-term surgical outcomes of the inverted internal limiting membrane flap technique in highly myopic macular hole retinal detachment. Graefes Arch Clin Exp Ophthalmol. 2017;255(6):1101–6. 10.1007/s00417-017-3614-0 28220252

[19] Michalewska Z, Michalewski J, Dulczewska-Cichecka K, Nawrocki J (2014). Inverted internal limiting membrane flap technique for surgical repair of myopic macular holes. Retina.

[20] Hozo SP, Djulbegovic B, Hozo I (2005). Estimating the mean and variance from the median, range, and the size of a sample. BMC Med Res Methodol.

[21] Ito Y, Terasaki H, Takahashi A, Yamakoshi T, Kondo M, Nakamura M (2005). Dissociated optic nerve fiber layer appearance after internal limitingmembrane peeling for idiopathic macular holes. Ophthalmology.

[22] Matsumura T, Takamura Y, Tomomatsu T (2016). Comparison of the inverted internal limiting membrane flap technique and the internal limiting membrane peeling for macular hole with retinal detachment. PLoS One.

[23] Baba R, Wakabayashi Y, Umazume K (2017). Efficacy of the inverted internal limiting membrane flap technique with vitrectomy for retinal detachment associated with myopic macular holes. Retina.

[24] Sasaki H, Shiono A, Kogo J (2017). Inverted internal limiting membrane flap technique as a useful procedure for macular hole-associated retinal detachment in highly myopic eyes. Eye (Lond).

[25] Chen SN, Yang CM. Inverted Internal Limiting Membrane Insertion for Macular Hole-Associated Retinal Detachment in High Myopia. Am J Ophthalmol. 2016;162:99–106.e1. 10.1016/j.ajo.2015.11.013 26582311

[26] Spiteri CK, Lois N, Scott NW (2014). Vitrectomy with internal limiting membrane peeling versus no peeling for idiopathic full-thickness macular hole. Ophthalmology.

[27] Kuriyama S, Hayashi H, Jingami Y, Kuramoto N, Akita J, Matsumoto M. Efficacy of inverted internal limiting membrane flap technique for the treatment of macular hole in high myopia. Am J Ophthalmol. 2013;156:125–131.e1. 10.1016/j.ajo.2013.02.014 23622567

[28] Madreperla SA, Geiger GL, Funata M, de la Cruz Z, Green WR (1994). Clinicopathologic correlation of a macular hole treated by cortical vitreous peeling and gas tamponade. Ophthalmology.

[29] Nishimura A, Kimura M, Saito Y, Sugiyama K (2011). Efficacy of primary silicone oil tamponade for the treatment of retinal detachment caused by macular hole in high myopia. Am J Ophthalmol.

[30] Nadal J, Verdaguer P, Canut MI (2012). Treatment of retinal detachment secondary to macular hole in high myopia: vitrectomy with dissection of the inner limiting membrane to the edge of the staphyloma and long-term tamponade. Retina.

[31] Modi A, Giridhar A, Gopalakrishnan M (2017). Spectral domain optical coherence tomography-based microstructural analysis of retinal architecture post internal limiting membrane peeling for surgery of idiopathic macular hole repair. Retina.

[32] Hayashi H, Kuriyama S (2014). Foveal microstructure in macular holes surgically closed by inverted internal limiting membrane flap technique. Retina.

[33] Lai CC, Chen YP, Wang NK (2015). Vitrectomy with internal limiting membrane repositioning and autologous blood for macular hole retinal detachment in highly myopic eyes. Ophthalmology.

[34] Casini G, Mura M, Figus M, et al. Inverted internal limiting membrane flap technique for macular hole surgery without extra manipulation of the flap. Retina 2017;doi:10.1097/IAE.0000000000001470. 10.1097/IAE.0000000000001470 28129215

[35] Michalewska Z, Michalewski J, Dulczewska-Cichecka K, Adelman RA, Nawrocki J (2015). Temporal inverted internal limiting membrane flap technique versus classic inverted internal limiting membrane flap technique: a comparative study. Retina.

[36] Mylonas G, Sacu S, Deak G, et al. Macular edema following cataract surgery in eyes with previous 23-gauge vitrectomy and peeling of the internal limiting membrane. Am J Ophthalmol. 2013;155:253–259.e2. 10.1016/j.ajo.2012.07.013 23036567

